# The Impact of Motor Axon Misdirection and Attrition on Behavioral Deficit Following Experimental Nerve Injuries

**DOI:** 10.1371/journal.pone.0082546

**Published:** 2013-11-25

**Authors:** Jacob Daniel de Villiers Alant, Ferry Senjaya, Aleksandra Ivanovic, Joanne Forden, Antos Shakhbazau, Rajiv Midha

**Affiliations:** 1 Department of Clinical Neurosciences, Hotchkiss Brain Institute, University of Calgary, Calgary, Alberta, Canada; 2 Department of Neuroscience, Hotchkiss Brain Institute, University of Calgary, Calgary, Alberta, Canada; University of Florida, United States of America

## Abstract

Peripheral nerve transection and neuroma-in-continuity injuries are associated with permanent functional deficits, often despite successful end-organ reinnervation. Axonal misdirection with non-specific reinnervation, frustrated regeneration and axonal attrition are believed to be among the anatomical substrates that underlie the poor functional recovery associated with these devastating injuries. Yet, functional deficits associated with axonal misdirection in experimental neuroma-in-continuity injuries have not yet been studied. We hypothesized that experimental neuroma-in-continuity injuries would result in motor axon misdirection and attrition with proportional persistent functional deficits. The femoral nerve misdirection model was exploited to assess major motor pathway misdirection and axonal attrition over a spectrum of experimental nerve injuries, with neuroma-in-continuity injuries simulated by the combination of compression and traction forces in 42 male rats. Sciatic nerve injuries were employed in an additional 42 rats, to evaluate the contribution of axonal misdirection to locomotor deficits by a ladder rung task up to 12 weeks. Retrograde motor neuron labeling techniques were utilized to determine the degree of axonal misdirection and attrition. Characteristic histological neuroma-in-continuity features were demonstrated in the neuroma-in-continuity groups and poor functional recovery was seen despite successful nerve regeneration and muscle reinnervation. Good positive and negative correlations were observed respectively between axonal misdirection (p<.0001, r^2^=.67), motor neuron counts (attrition) (p<.0001, r^2^=.69) and final functional deficits. We demonstrate prominent motor axon misdirection and attrition in neuroma-in-continuity and transection injuries of mixed motor nerves that contribute to the long-term functional deficits. Although widely accepted in theory, to our knowledge, this is the first experimental evidence to convincingly demonstrate these correlations with data inclusive of the neuroma-in-continuity spectrum. This work emphasizes the need to focus on strategies that promote both robust and accurate nerve regeneration to optimize functional recovery. It also demonstrates that clinically relevant neuroma-in-continuity injuries can now also be subjected to experimental investigation.

## Introduction

Axonal misdirection is believed to play a significant role in poor functional recovery seen after severe nerve injuries [1–3]. Although widely accepted, this relationship is not yet established by experimental evidence to demonstrate a positive correlation between axonal misdirection and behavioral deficit following sub-transection injuries [4].

Axonal misdirection is strictly not expected to follow pure axonotmetic (Sunderland Grade 2 or crush) injuries in which full functional recovery usually occurs [5,6]. This is thought to be because of preservation of endoneurial connective tissue integrity, which helps axons to regenerate accurately and efficiently to the original targets. In contrast, unrepaired transection injuries (Sunderland Grade 5) are not expected to recover any significant function, although rodents are known to be able to regenerate axons across substantial transection gaps [7,8]. Unrepaired transection injuries should represent the extreme of axonal misdirection, provided that the regeneration gap is bridged. 

In order to effectively investigate the relationship between axonal misdirection and functional deficit, the gap in experimental data between minimal and extreme nerve injuries needed to be bridged. These intermediate sub-transection (Sunderland Grade: 3 - endoneurial and 4: - endo- and perineurial disruption) injuries that result in neuroma-in-continuity (NIC) formation are the most challenging clinically and have previously been elusive to experimental investigation because of the lack of an appropriate animal model. Recently a clinically relevant traumatic NIC model was presented that showed some promise to bridge this gap [9]. Although sciatic nerve NIC injuries were associated with functional deficits discernable from crush injuries, this model was not mechanistically validated by the unequivocal demonstration of quantitative data to support the histological and functional findings.

We hypothesized that experimental NIC injuries would result in motor axon misdirection and attrition with persistent functional deficits. We show how the NIC, femoral and sciatic nerve injury models were exploited to demonstrate 1) axonal misdirection and attrition of motor neurons with motor pathway projections in NIC injuries, similar to transection injuries; 2) for the first time, a direct correlation between the degree of motor axon misdirection and behavioral deficit, with inclusion of the NIC injury spectrum; 3) a negative correlation between functional recovery and the degree of attrition of motor neurons projecting into motor pathways. We hereby demonstrate how this small animal NIC model (with some refinement), can help to expose otherwise elusive substrates of nervous system injuries to experimental investigation and possible therapeutic manipulation.

## Materials and Methods

### Ethics Statement

Male Lewis rats were used in these experiments (Charles River Laboratories International Inc., St-Constant, QC, Canada). The study protocol was approved by the University of Calgary Animal Care Committee and adhered strictly to the Canadian Council on Animal Care guidelines (protocol M08124).

### General animal care

All efforts were made to minimize suffering and animals were maintained in a temperature and humidity controlled environment with standard rat chow (Purina, Mississauga, ON, Canada), water *ad libitum* and a 12:12h light:dark cycle. Surgical procedures were performed under inhalation anesthetic (Isoflurane, Pharmaceutical Partners of Canada Inc., Richmond Hill, ON, Canada) using standard microsurgical and aseptic technique and an operating microscope. Buprenorphine (0.03mg/kg) subcutaneous injections followed by jello with buprenorphine were used for peri- and post-operative analgesia. Surgical procedures were well tolerated by all animals, with no complications observed. Animals were sacrificed at study termination, under deep inhalation anesthesia with intra-peritoneal Somnitol (Bimeda-MTC, Cambridge, ON, Canada) followed by trans-cardiac perfusion with saline and 2% paraformaldehyde. 

### Instruments and nerve characteristics

A malleus nipper (MN) (16149-11, Fine Science Tools Inc., North Vancouver, BC, Canada) was modified to exert uniform compression and an adjustable stop mounted to the handles to enable instrument calibration with a thin load cell (Figure S1; Text S1). It has been suggested that the addition of traction to a compression force would aid the reproduction of NIC injuries [9]. We employed traction by applying 3-second compression (with the malleus nipper) in combination with simultaneous 50g-traction with a spring scale (100x1g, AMW-PEN100, American Weigh Sales Inc., Norcross, GA, USA) hooked around the nerve, pulling orthogonal to the native nerve course. During pilot experiments this technique produced clear histological features of uniform NIC formation in sciatic and femoral nerves at 5 days (Figure S2). With threshold data collected during pilot experiments, sub-transection settings on the MN were selected with the aim to reproduce NIC injuries in groups of animals by using the “tight” nerve diameter range of target nerves in these experimental groups (Figure S1; Text S1). 

### Femoral nerve experiments

Selective and variable disruption of the internal nerve architecture of a nerve would potentially result in proportional axonal misdirection and attrition. We first set out to investigate to what degree we could induce misdirection within an injured nerve without disruption of its gross epineurial continuity. We applied the newly developed NIC injury model to the femoral nerve misdirection model, which is favorably suited and established to investigate axonal misdirection [10]. The rodent femoral nerve terminates in a motor (quadriceps) and a cutaneous division (saphenous and thigh skin) of similar size [10]. Following a more proximal injury, the motor neurons with axons that are misdirected into the cutaneous division can be back labeled by various techniques to estimate the degree of misdirection [11]. 

**Figure 1:**
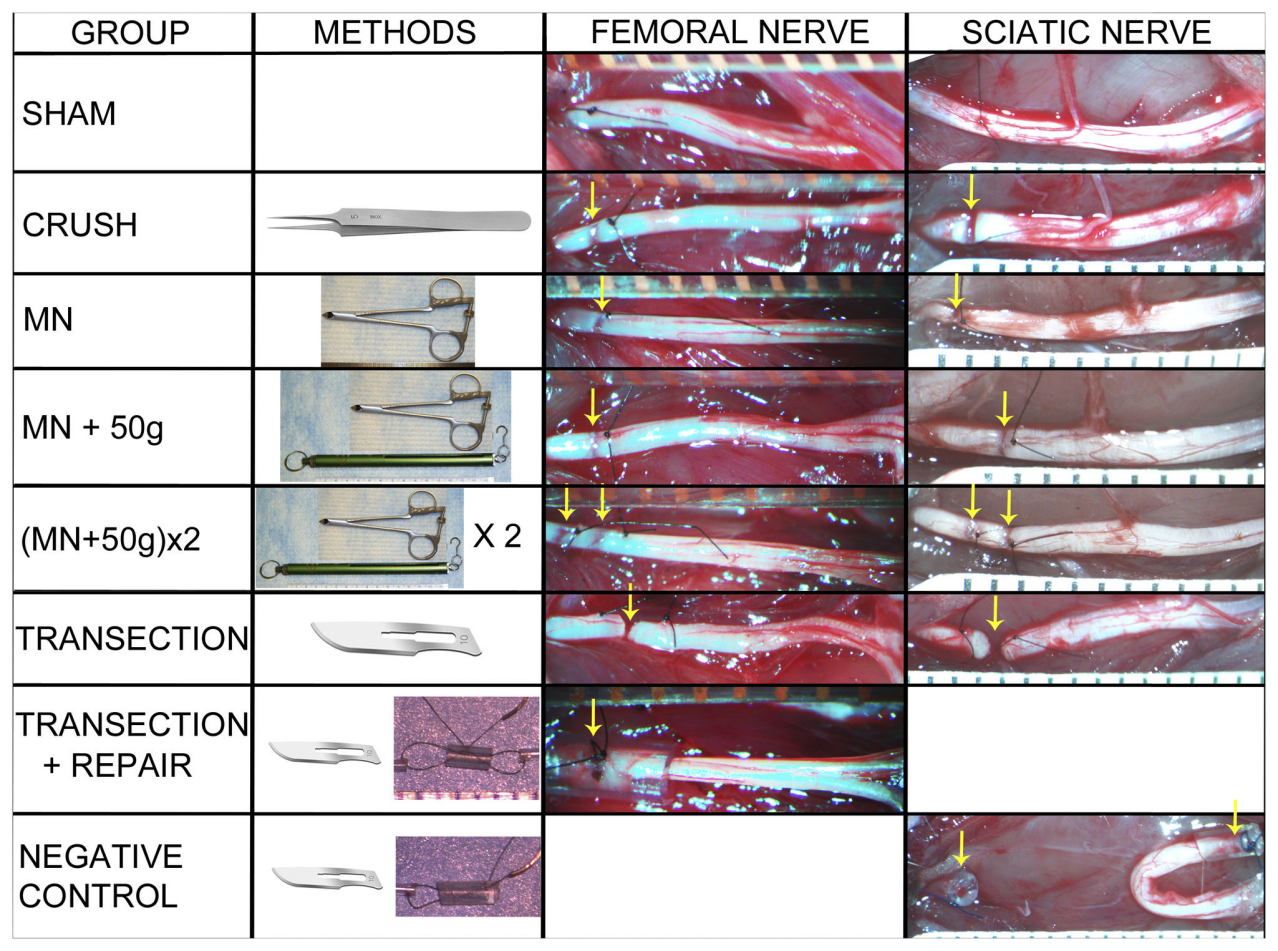
Experimental groups. Representative pictures of injury zones (arrows) are shown for each of the femoral and sciatic nerve experimental groups, proximal ends to the left. SHAM – nerve exposure and suture marking only; CRUSH – simple 30-second jeweler’s forceps crush; MN – single 3-second compression using sub-transection force; MN+50g – single MN compression combined with 50g traction force; (MN+50g)x2 – two MN+50g injuries made in tandem; TRANSECTION – sharp transection without repair; TRANSECTION+REPAIR – intra-tubular repair of the transected femoral nerves; NEGATIVE CONTROL – transection, capping and back-reflection of sciatic nerve ends as negative control.

42 rats, weighing 250-300g were randomized into one of seven groups of six rats each for left femoral nerve surgeries (Figure 1). At 28 days, fast blue (FB) (Polysciences Inc., Warrington, PA, USA) and 1,1'-dioctadecyl-3,3,3',3'-tetramethyl-indocarbo-cyanine perchlorate (Di-I) (Invitrogen, Molecular Probes, Eugene, OR, USA) were respectively applied distally, to the main motor (FB) and cutaneous (Di-I) divisions of the nerves for retrograde labeling of spinal cord motor neurons. Distal motor division nerve segments were also harvested at this time for histomorphometry. 13 days later spinal cords and femoral nerves were harvested for longitudinal cryostat sectioning, counting of fluorescently labeled neurons and histological evaluation of injury zones. 

For all the injury groups the femoral nerve was exposed in the groin up to the origin of the iliacus nerve by division of the inguinal ligament. The target injury site was marked with a mesoneurial 9.0 nylon (Ethilon, Johnson & Johnson Medical, Markham, ON, Canada) suture, 7-7.5mm proximal to the femoral nerve division point, and the “tight” nerve diameter measured at this site with electronic calipers. 

In the ***Sham*_*f*_** (“**_*f*_**” denoting femoral nerve experiments) group, wounds were closed after the marking suture placement. Crush injuries were included as established axonotmetic injuries for comparison [12]. Ideally no misdirection or long-term functional deficit should result from these injuries, although misdirection and extrafascicular regeneration have been previously described in “crush” injuries [13,14]. Simple 30-second ***Crush*_*f*_** injuries were made just proximal to the marking suture with jeweler’s forceps (Dumont #5, Fine Surgical Tools Inc., North Vancouver, BC, Canada). In the ***Transection*_*f*_** group, marking sutures were placed at 7 and 8mm (1mm apart) proximal to the femoral division point and the femoral nerve sharply transected, midway between the sutures (Figure 1). The ends were left to retract *in situ* and the wounds closed. This was to simulate unrepaired Sunderland Grade 5 injuries, as the most severe category of peripheral nerve injury. 

Femoral nerve diameters of the above groups were used to select an appropriate sub-transection setting (3690g) (representative of this cohort of animals) on the MN using data of pilot experiments (Figure S3; Text S1). Three injury paradigms were used with the aim to reproduce NIC injuries (Figure 1). For the ***MN*_*f*_** group, a single 3-second injury was made just proximal to the single marking suture (no traction). The spring scale was hooked around the femoral nerve to apply 50g of traction, when a 3-second MN injury (same 3690g setting) was made just proximal to the suture in the ***MN*+50*g*_*f*_** group. Traction was released after the 3-second injury. For the (***MN+50g***)***x2*_*f*_** group (“double” femoral NIC-injury group), two marking sutures were placed (7 and 8mm proximal to division point), and the above procedure repeated, first the proximal then the distal injury, with brief release of the traction between injuries. This group was included in an attempt to maximize disruption of the internal nerve architecture at the selected force setting. The ***Transection+Repair*_*f*_** group was included to simulate immediate direct coaptation of Grade 5 injuries as a “gold standard” therapeutic strategy for reference. Here the nerve was transected at 7.5mm as above (without marking sutures). For improved ease and security of the repair, nerve ends were approximated within a 3mm long silicone tube with 9.0 nylon (0.64mm internal diameter, Silastic Laboratory Tubing, Dow Corning Corporation, Midland, MI, USA). The mean “tight” nerve diameter of the femoral nerve at the target injury site as measured at the initial surgery was 0.54mm (range 0.46 - 0.6mm).

#### Retrograde labeling, specimen processing and microscopy (femoral nerve experiments)

At 28 days, femoral nerves were re-exposed distal to the inguinal ligament. Femoral nerve motor division nerve segments (1-2mm) were harvested just distal to the point where the nerves were transected for FB application (see below). These segments were fixed in 2.5% glutaraldehyde, post fixed in osmium tetroxide, Epon resin-embedded, 1µm semi-thin transverse sections cut (LKB 8800 Ultratome III, Bromma, Stockholm, Sweden) and stained with toluidine blue for histomorphometrical analysis. Five fields of view per section were photographed at high power light microscopy (400x; Olympus BX51) for analysis. Images were digitized with a Wacom Intuos3 digitizing tablet (Vancouver, WA, USA) and analyzed using Image Pro Plus software (Media Cybernetics, Bethesda, MD, USA). Axon and myelin measurements were used to calculate the average G-ratios (axon diameter/fiber diameter) and percentage of neural tissue (fiber area/intrafascicular area) for each nerve. 

The distal motor and cutaneous divisions were “capped” with small silicone caps (3mm long, 0.64 mm ID), carefully prefilled with a small amount of FB or Di-I crystals, respectively, and secured with 9.0 nylon [15].

Femoral nerve injury zones were harvested immediately prior to perfusion (28+13= 41 days post injury), fixed in 10% formalin, cryoprotected in 30% sucrose-phosphate buffered saline and then embedded in optimal cutting temperature (OCT) compound (Sakura Fine Technical Co., Torrance, CA, USA). The distance between marking-sutures in the ***Transection*_*f*_** group were recorded after careful exploration of the injury zones. Serial 8µm cryotome (Leica CM1900; Leica Microsystems Inc., Richmond Hill, ON, Canada) longitudinal sections were cut at -18°C and mounted onto Superfrost Plus slides (Fisher Scientific, Ottawa, ON, Canada). Selected sections were double stained for NF (NF200, 1:600 dilution; Sigma-Aldrich) and rhodamine phalloidin (Invitrogen, Life Technologies Inc., Burlington, ON, Canada) (f-actin stain to highlight perineurium) according to supplier protocols. The examiner was blinded to the experimental group allocation until sections were examined and injury zones photographed with a fluorescence microscope (Olympus BX51, Olympus America Inc., Center Valley, PA, USA) using appropriate filters. 

13 days after tracer application and just after harvesting of the femoral nerve injury zones, the animals were perfused. Lumbar spinal cords (T12 to conus) were harvested, cryoprotected in paraformaldehyde and sucrose solution and embedded in OCT for longitudinal (coronal) 45µm cryostat sectioning. All sections through the ventral grey horns were collected and mounted on glass slides for counting of the labeled motor neurons. With the fluorescent microscope and appropriate filters, pictomicrographs were taken for counting of total (the sum of all labeled neurons counted on all the slides of each spinal cord) FB, total Di-I and double labeled motor neurons (visible on superimposed pictures) by a blinded observer. Double labeled neurons were included in both FB and Di-I counts. Two spinal cord specimens were damaged during cutting so that not all consecutive sections could be assessed and these two were excluded from analysis, one each in the following groups: (***MN+50g***)***x2*_*f*_** and ***Transection+Repair***_f_.

### Sciatic nerve experiments

Although functional recovery following mouse femoral nerve injuries has been successfully assessed before, the sciatic nerve model is most widely employed for behavioral outcome measures in rodents [16–18]. We therefore returned to the well-established sciatic nerve model to assess the relative impact that different degrees of nerve injury (and the associated misdirection and attrition investigated in the simpler femoral nerve model) have on integrated functional recovery. Assessment of axonal misdirection is however more challenging in the sciatic nerve model. We employed a motor neuron sampling technique to provide a conservative misdirection assessment (Text S1). This allowed us to investigate the correlation between axonal misdirection (and attrition) and functional recovery within an established behavioral assessment model.

42 rats, weighing 300-350g, were randomized into one of seven similar groups of six rats each for right sciatic nerve surgeries (Figure 1). Skilled locomotion with horizontal ladder rung data was collected at baseline and serially up to 12 weeks. After 12 weeks, FB and Di-I were respectively applied distally, to the medial gastrocnemius (MG) and sural nerves for retrograde labeling of spinal cord motor neurons. 13 days later spinal cords were harvested and processed for counting of fluorescently labeled neurons and right tibialis anterior muscles were harvested for wet muscle weight.

For all the sciatic nerve injury groups the right sciatic nerve was exposed from where it emerges over the external obturator muscle from the sciatic notch, to the trifurcation above the popliteal fossa. The target injury site was marked with a mesoneurial 9.0 nylon suture, 9.5- 10.5mm proximal to the sciatic nerve trifurcation point, and the “tight” nerve diameter measured at this site.

 In the ***Sham*_*s*_** (“**_*s*_**” denoting sciatic nerve experiments) surgery group, wounds were closed after the marking suture placement. Simple 30-second ***Crush*_*s*_** injuries were made just proximal to the marking suture with jeweler’s forceps. In the ***Transection*_*s*_** group, marking sutures were placed at 9.5 and 10.5mm (1mm apart) proximal to the sciatic trifurcation and the nerve sharply transected midway between the sutures (Figure 1). The ends were left to retract *in situ* and the wounds closed. The ***NegativeControl*_*s*_** group was added mainly for the behavioral component of the study. Here the nerve was transected at 10mm as above and the ends capped with tight-fitting 3-mm long silicone tubes (0.64mm ID, one end pre-sealed with silicone) and the distal stump reflected to a subcutaneous location, to deter spontaneous regeneration. 

Sciatic nerve diameters of these groups were used to select the setting of 4860g on the MN for the three NIC groups (Figure S3). In the ***MN*_*s*_** group, a single 3-second injury was made just proximal to a marking suture at 10mm (no traction). The spring scale was hooked around the sciatic nerve to apply 50g of traction, when a 3-second MN injury was made just proximal to the marking suture in the ***MN*+50*g*_*s*_** group. Traction was released after the 3-second injury. In the (***MN+50g***)***x2*_*s*_** group, two marking sutures were again used (at 9 and 10mm proximal to the trifurcation), and the above procedure repeated, first the proximal then the distal injury, with brief release of the traction between the injuries. The mean “tight” nerve diameter of the sciatic nerve at the target injury site as measured at the initial surgery was 0.91mm (range 0.78 - 1.01mm). 

#### Retrograde labeling, specimen processing and microscopy (sciatic nerve experiments)

After the 12-week skilled locomotion assessment, MG and sural nerves were exposed in the popliteal fossa. FB and Di-I was applied to the right MG and sural nerves respectively, by capping the severed nerves with small, prefilled silicone caps (2.5 mm long, 0.51 mm ID). 

**Figure 2:**
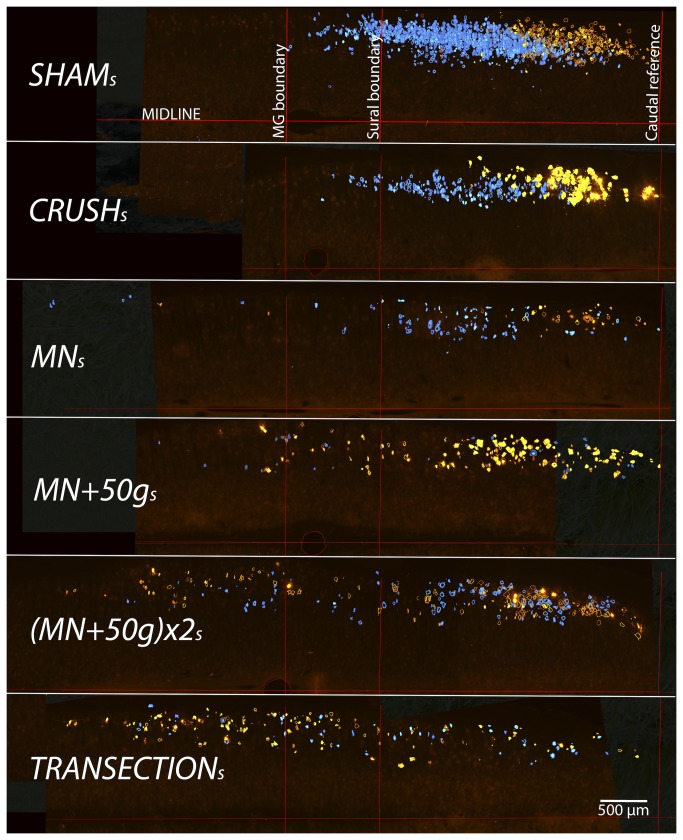
Selective sciatic motor neuron labeling for assessment of relative axonal misdirection. For demonstration purposes, representative coronal cut hemi-cord examples (stacks of all spinal cord sections of a single animal from each group) show the disorganization of labeled neurons after more severe nerve injuries, compared to *Sham*_***s***_ (stack of all 6 sham cords used to define the reference boundaries). FB (blue) or Di-I (yellow) labeled cells outside the reference bounds have axons misdirected respectively to the MG or sural nerves. Caudal sciatic pool boundaries were aligned to overlay the *Sham*_***s***_ group pool reference grid for the determination of motor neurons with misdirected axons (500µm scale bar).

13 days after tracer application and just after harvest and weighing of the right tibialis anterior muscles, the animals were perfused and the lumbar (T12- conus) spinal cords harvested. The distance between marking-sutures in the ***Transection*_*s*_** group were also recorded after careful exploration of the injury zones. A midline reference pin was placed at the L5/L6 dorsal rootlet junction and cords embedded, cut, mounted and photographed as with the femoral experiments described above. For each spinal cord, digital pictures of serial sections were positioned along the same longitudinal axis (midline of spinal cord) with the pinholes aligned on superimposed pictures (Adobe Photoshop Elements 8, Adobe Systems Incorporated, San Jose, CA, USA). For each animal, the most caudally labeled cell among all the sections was used to identify the caudal reference. Using the picture compilations of the six animals in the ***Sham*_*s*_** group, the maximum rostro-caudal extent of the MG and sural nerve motor neuron pools were determined and used as reference boundaries for all other groups (Figure 2). While blinded, all blue (MG) and yellow (sural) labeled motor neurons were counted on each section (and added together for each spinal cord) with an overlay reference grid and scored as either “in-” or “out-” side the reference MG or sural longitudinal boundaries. No attempt was made to correct for double counted motor neurons. The relative percentage of motor neurons with misdirected axonal projections was calculated for MG and sural samples (%misdirection MG = FB out/FB total x 100; %misdirection sural = Di-I out/Di-I total x 100). The average percentage misdirection for each animal and group was also calculated as the average between the misdirection results of the MG and sural nerve sampling techniques.

#### Behavior (sciatic nerve experiments)

The horizontal ladder rung task has been successfully used previously to assess return of skilled locomotor behavior following hind limb nerve injuries in rats [9,15,19]. Animals were trained to cross a horizontally placed ladder prior to surgical intervention. The apparatus consists of sidewalls made of clear plexiglass (1m long, 20cm high) and metal rungs (3mm diameter), which are inserted 1cm from the bottom of the plexiglass and can be spaced 1cm or more apart. During testing, an irregular pattern of the rungs (1-3cm apart) was changed from trial to trial in order to prevent the animals from learning the spacing pattern. Five satisfactory runs were used for each animal and time point, with a satisfactory run consisting of the animal travelling across the beam uninterrupted at a constant velocity. A mirror was placed at a 45° angle below the ladder so that the rats could be video-recorded with a simultaneous lateral and a ventral view. Steps with the right hind limbs were scored as a correct or an incorrect (total miss or deep slip) step [19]. A slip ratio (%) was calculated as the number of right hind limb slips per total number of right hind limb steps. Recordings were analyzed frame-by-frame by a trained observer, blinded to the experimental conditions. Data were analyzed for baseline and weeks 2, 6, 10 and 12 following surgery.

### Statistical analysis

Data were analyzed with GraphPad Prism 4 (GraphPad Software, San Diego, CA, USA) software using one-way ANOVA and *post hoc* Tukey’s HSD test to compare results between groups. For behavioral data, repeated measures ANOVA with *post hoc* Tukey’s HSD test were used for pairwise comparisons where appropriate. Linear correlations (Pearson) were investigated between final functional outcome (12-week slip ratio) and average percentage misdirection as well as the total number of motor neurons projecting into the main motor (MG) branch for all individual animals with paired results. Statistical significance was accepted at the level of p<0.05, with results presented as the mean ± SEM. 

## Results

### The “double” femoral NIC-injury group had histomorphometry similar to neurotmetic groups

**Figure 3:**
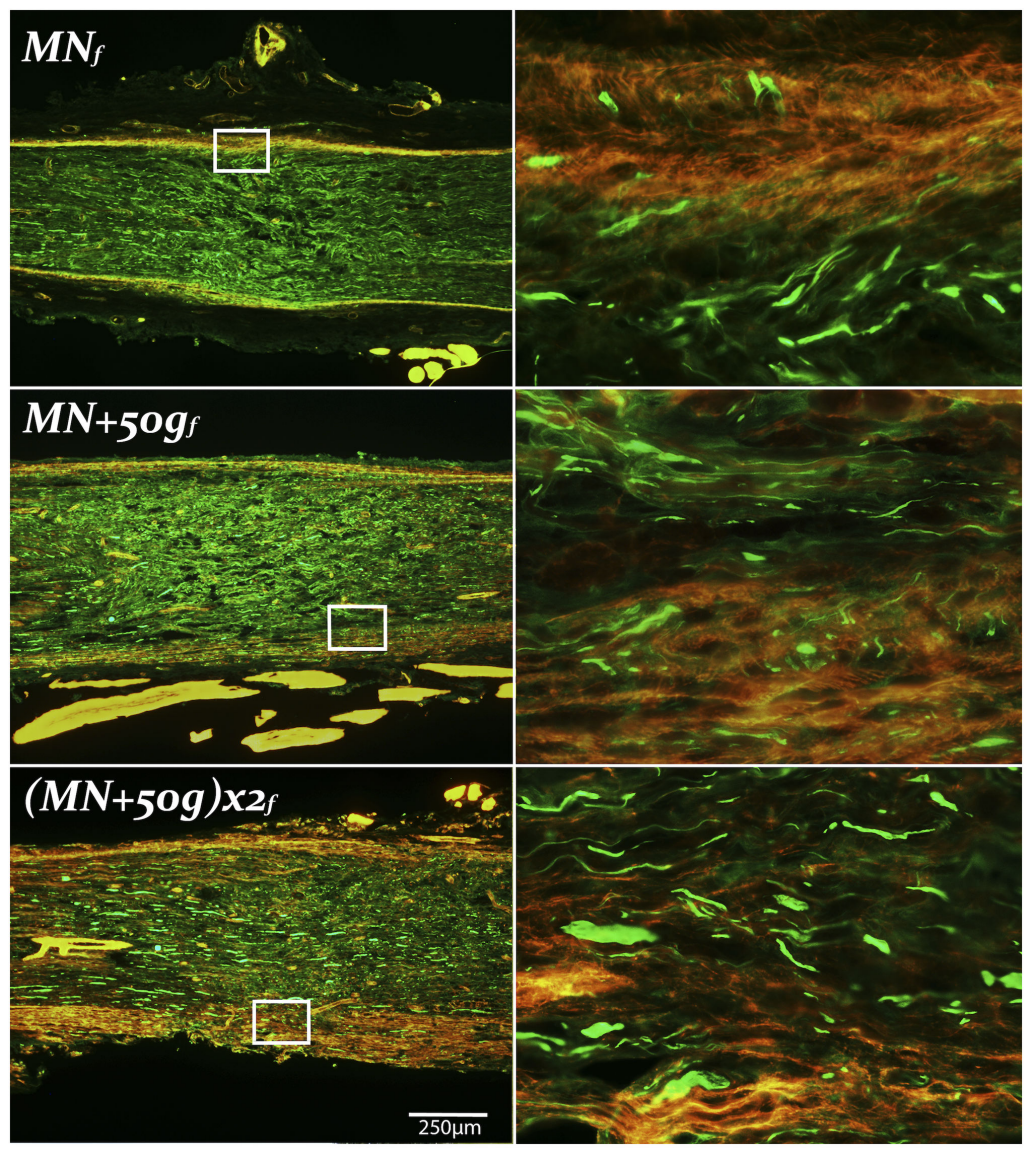
Neuroma formation in femoral nerve NIC groups. Representative longitudinal femoral nerve sections of the experimental NIC groups, with magnified areas demonstrating extrafascicular regeneration on the right. *MN*_*f*_ injury zones showed the least, and (MN+50g)***x2***_***f***_ (proximal injury) the most prominent NIC features. Proximal ends to the left, NF 200 in green, Rhodamine Phalloidin (f-actin) in red (250µm scale bar).

Histology of the injury zones harvested at 41 days after injuries were assessed for signs of aberrant intra- and extrafascicular axonal regeneration by an observer blinded to the group allocation. The rhodamine phalloidin f-actin stain was useful to delineate the perineurium in order to detect perineurial infiltration and extrafascicular regeneration of NF strained axons. Although features of NIC were identified in all three NIC injury groups, this was not quantifiable with a validated method (Figure 3). The mean distance between the two marking sutures (initially 1mm apart) in the ***Transection*_*f*_** group at harvest was 3.45mm (range 2.0 - 5.15mm).

**Figure 4:**
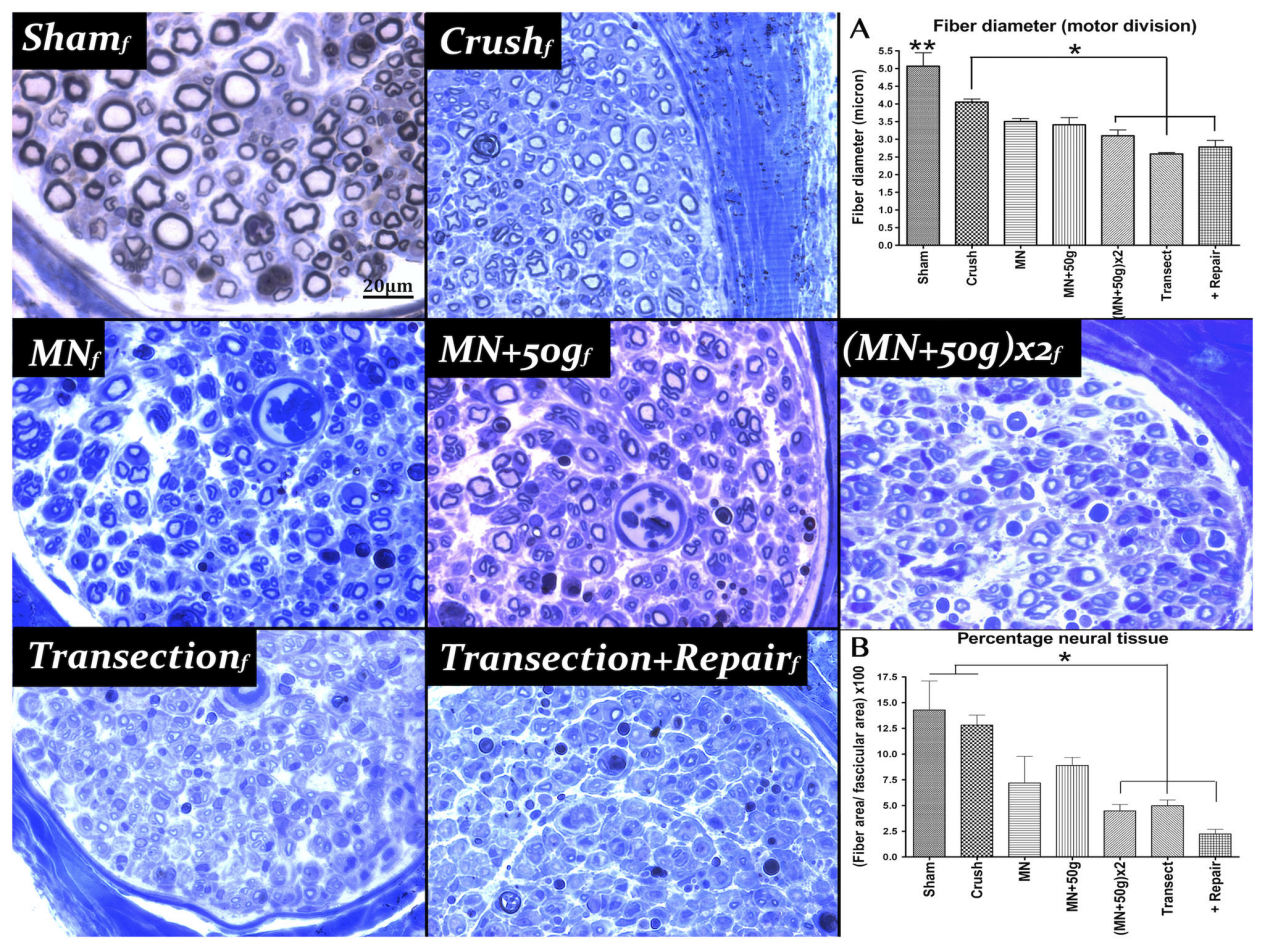
Femoral nerve motor division histomorphometry. Representative semi-thin transverse sections of the motor division of each femoral nerve group stained with toluidine blue. A) Fiber diameters in two NIC groups were not significantly different from *Crush*_***f***_ (n=6), unlike the (MN+50g)***x2***_***f***_ (n=5), *Transection*_***f***_ (n=5) and *Transection+Repair*_***f***_ (n=6) groups (*), although all were different from *Sham*_***f***_ (**). B) Percentage neural tissue in the (MN+50g)***x2***_***f***_ group showed statistically significant differences compared to the *Sham*_***f***_ (n=6) and *Crush*_***f***_ groups, similar to the *Transection*_***f***_ and *Transection+Repair*_***f***_ groups. *MN*_*f*_ (n=3); *MN*+50*g*_*f*_ (n=4); 20µm scale bar (* and ** p<0.05).

Femoral nerve motor division segments that were harvested distal to the FB application site at 28 days that were suitable for histomorphometrical evaluation, had similar G-ratios although statistically significant differences (*F*(6, 28)=3.23, *p*=.015) were shown comparing ***Sham*_*f*_** with ***Transection*_*f*_** and ***Transection+Repair*_*f*_** groups (*p*<.05) (Table 1). Fiber diameters were significantly different between groups (*F*(6, 28)=17.19, *p*<.0001) with the ***Sham*_*f*_** group fibers larger than all other groups (*p*<.05). ***Crush*_*f*_** group fibers were also larger than (***MN+50g***)***x2*_*f*_**, ***Transection*_*f*_** and ***Transection+Repair*_*f*_** groups (*p*<.05) (Figure 4A). Percentage neural tissue (*F*(6, 28)=9.62, *p*<.0001) in the ***Sham*_*f*_** and ***Crush*_*f*_** groups were significantly larger than the (***MN+50g***)***x2*_*f*_**, ***Transection*_*f*_** and ***Transection+Repair*_*f*_** groups (*p*<.05) (Figure 4B). 

**Table 1:** Femoral nerve (motor division) histomorphometrical and retrograde labeling results.

**Group (femoral)**	**Sham_f_**	**Crush_f_**	**MN_f_**	**MN+50g_f_**	**(MN+50g)x2_f_**	**Transection_f_**	**Transection+Repair_f_**
**G-ratio**	0.71 ± 0.01	0.68 ± 0.01	0.66 ± 0.01	0.68 ± 0.01	0.69 ± 0.01	0.66 ± 0.02	0.66 ± 0.01
**Fiber diameter** (µm)	6.07 ± 0.38	4.05 ± 0.09	3.5 ± 0.09	3.41 ± 0.2	3.1 ± 0.17	2.58 ± 0.04	2.78 ± 0.19
**Neural tissue (%)**	14.28 ± 2.82	12.82 ± 0.98	7.19 ± 2.58	8.89 ± 0.78	4.47 ± 0.63	4.98 ± 0.55	2.24 ± 0.43
**FB (motor division)**	390.2 ± 30.15	420 ± 13.03	298.8 ±57.63	298.3 ±41.6	137.8 ± 40.06	162.4 ± 47.36	154.8 ± 26.78
**Di-I (cutaneous**)	0	0.5 ± 0.5	12.33 ± 5.4	14.67 ±12.9	53.25 ± 19.83	102.2 ± 19.4	50.8 ± 24.36
**Double (FB and Di-I)**	0	0	0.67 ± 0.49	1 ± 1	2.25 ± 2.25	9.6 ± 4.76	3.4 ± 1.54
**Total (FB + Di-I - Double)**	390.2 ± 30.15	420.5 ±12.66	310.5 ±60.55	312 ± 46.6	188.8 ± 53.5	255 ± 50.79	202.2 ± 48.58
**Misdirection (%)**	0	0.13 ±0.13	4.67 ± 2.1	3.73 ± 2.84	26.84 ± 7.68	45.2 ± 8.51	20.2 ± 6.57

### Axonal attrition and misdirection in the “double” femoral NIC-injury group were also similar to neurotmetic injuries

After 13 days, successful Di-I and FB motor neuron labeling occurred in all but three of 40 cords assessed (two of 42 were damaged). One of the ***Sham*_*f*_** group demonstrated aberrant Di-I labeling, likely due to leakage at the application site. Di-I labeling was unsuccessful in another two animals (***Transection*_*f*_**; (***MN+50g***)***x2*_*f*_** ) and these were excluded from analysis. The following group statistics were evaluated after unblinding: FB counts (motor neurons back-labeled via axons that regenerated down the distal motor division), Di-I counts (motor neurons back-labeled via axons misdirected down the distal cutaneous division), double label counts (motor neurons labeled by both dyes due to collateral axonal sprouting and regeneration into both motor and cutaneous divisions), total motor neurons labeled (FB + Di-I minus double label) and misdirection percentage (Di-I/Total x 100). 

**Figure 5:**
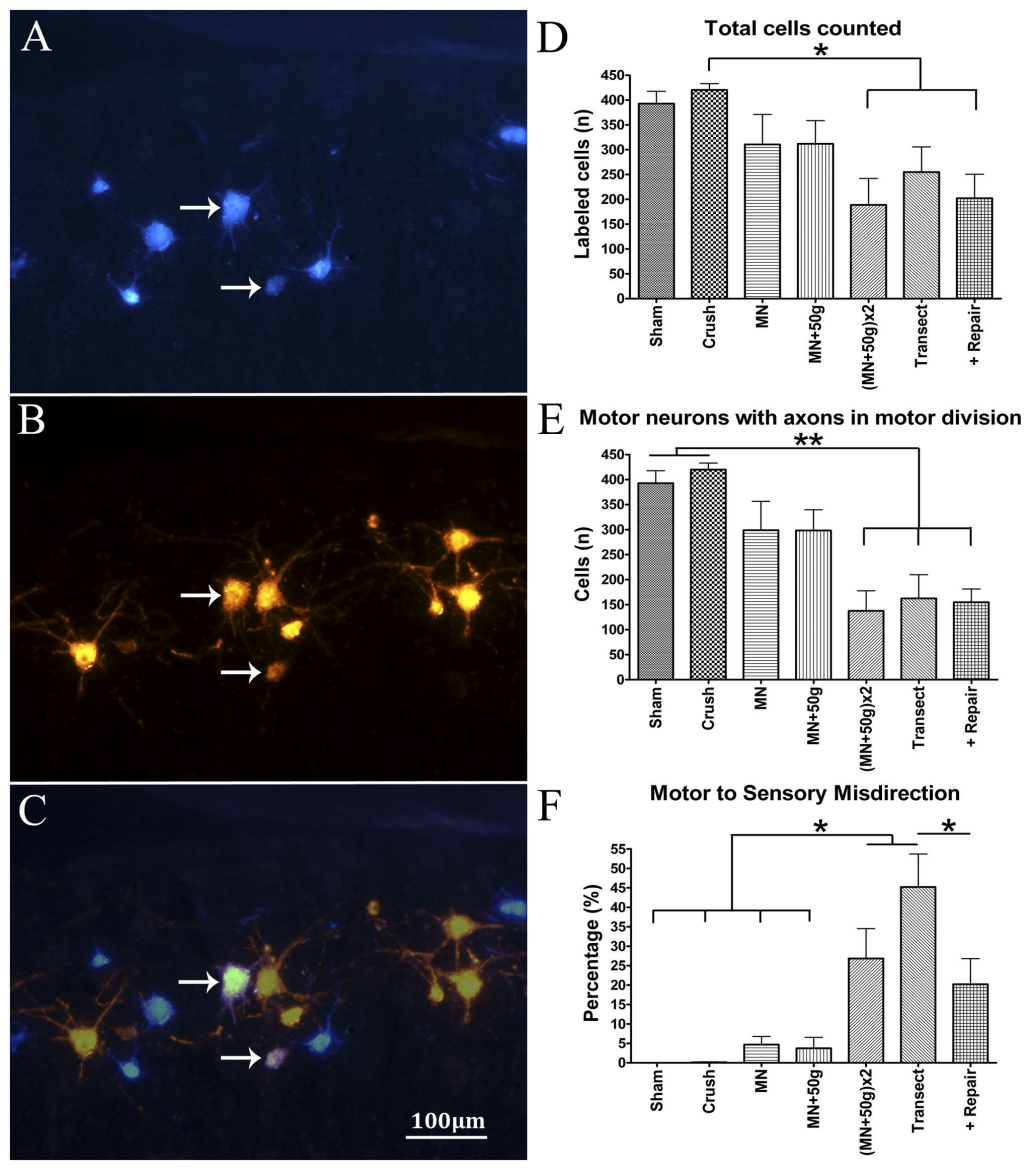
Femoral nerve motor neuron labeling results. Results of motor neuron labeling by FB (A) and Di-I (B) application to femoral nerve motor and cutaneous divisions, respectively. Double-labeled motor neurons (C) are illustrated by the arrows (merge A+B). The total MN counts (FB + Di-I minus double label) represent the overall degree of attrition of motor neurons that regenerated axons beyond the injury zone. The total *Crush*_***f***_ (n=6) group counts dominated over (MN+50g)***x2***_***f***_ (n=4) and *Transection+Repair*_***f***_ (n=5) groups with statistical significance (D). FB cell counts of the *Sham*_***f***_ (n=5) and *Crush*_***f***_ groups showed statistically significant differences compared to the (MN+50g)***x2***_***f***_, *Transection*_***f***_ (n=5) and *Transection+Repair*_***f***_ groups, indicating significant motor pathway attrition in the latter groups (E). Percentage motor axons misdirected to the cutaneous division (= Di-I labeled/ Total count x100) was the highest in the *Transection*_***f***_ group and together with the (MN+50g)***x2***_***f***_ group, had statistically significant differences from the Sham_***f***_, Crush_***f***_, *MN*_*f*_ (n=6) and *MN*+50*g*_*f*_ (n=6) groups. The *Transection*_***f***_ group had also significantly more misdirection compared to the *Transection+Repair*_***f***_ group (F); 100µm scale bar (*p<0.05;**p<0.01).

Total motor neurons labeled differed significantly between groups (*F*(6, 30)=3.57, *p*=.0087) (Table 1). As expected, practically only FB motor neurons were present in the ***Sham*_*f*_** and ***Crush*_*f*_** groups, because no axonal misdirection should be found in these groups. The highest counts were found in the ***Crush*_*f*_** group, significantly more than in the (***MN+50g***)***x2*_*f*_** and ***Transection+Repair*_*f*_** groups (*p*<.05) (Figure 5D). The FB counts represent the relative attrition of motor neurons with axons in the motor division of the femoral nerve. There were also significant differences in FB counts between the groups (*F*(6, 30)=8.11, *p*<.0001) (Figure 5E). The lowest number of FB labeled cells (most attrition) was found in the (***MN+50g***)***x2*_*f*_** group, similar to the ***Transection*_*f*_** and ***Transection+Repair*_*f*_** groups and significantly lower than the ***Sham*_*f*_** and ***Crush*_*f*_** groups (*p*<.01). 

The percentage axonal misdirection (Di-I/Total x 100) differed significantly between groups (*F*(6, 30)=13.22, *p*<.0001) (Figure 5F). Axonal misdirection was the highest in the ***Transection*_*f*_** group and together with the (***MN+50g***)***x2*_*f*_** group, had statistically significant differences from the ***Sham*_*f*_**, ***Crush*_*f*_**, ***MN*_*f*_** and ***MN*+50*g*_*f*_** groups (*p*<.05). The ***Transection*_*f*_** group had also significantly more misdirection compared to the ***Transection+Repair*_*f*_** group (*p*<.05), supporting the advantage of nerve repair. Although relatively few double-labeled cells were detected, groups also demonstrated some differences in double-label counts (*F*(6, 30)=2.98, *p*=.02). The ***Transection*** group had the most motor neurons with axonal processes in both major distal femoral divisions (Table 1). This was significantly more than in ***Sham*_*f*_**, ***Crush*_*f*_**, ***MN*_*f*_** and ***MN*+50*g*_*f*_** groups (*p*<.05). These results indicate that the double experimental NIC injuries (***(MN+50g***)***x2*_*f*_**) were similar to unrepaired Sunderland Grade 5 injuries (***Transection*_*f*_**) in terms of axonal attrition in the motor division and major pathway (motor to sensory) misdirection. The absence of traction did not appear to make a difference in these femoral nerve experiments (***MN*_*f*_** group), but inclusion of the (***MN+50g***)***x2*** group effectively captured more severe injuries.

### Muscle weights of sciatic transections injuries suggest impaired but partial reinnervation

**Figure 6:**
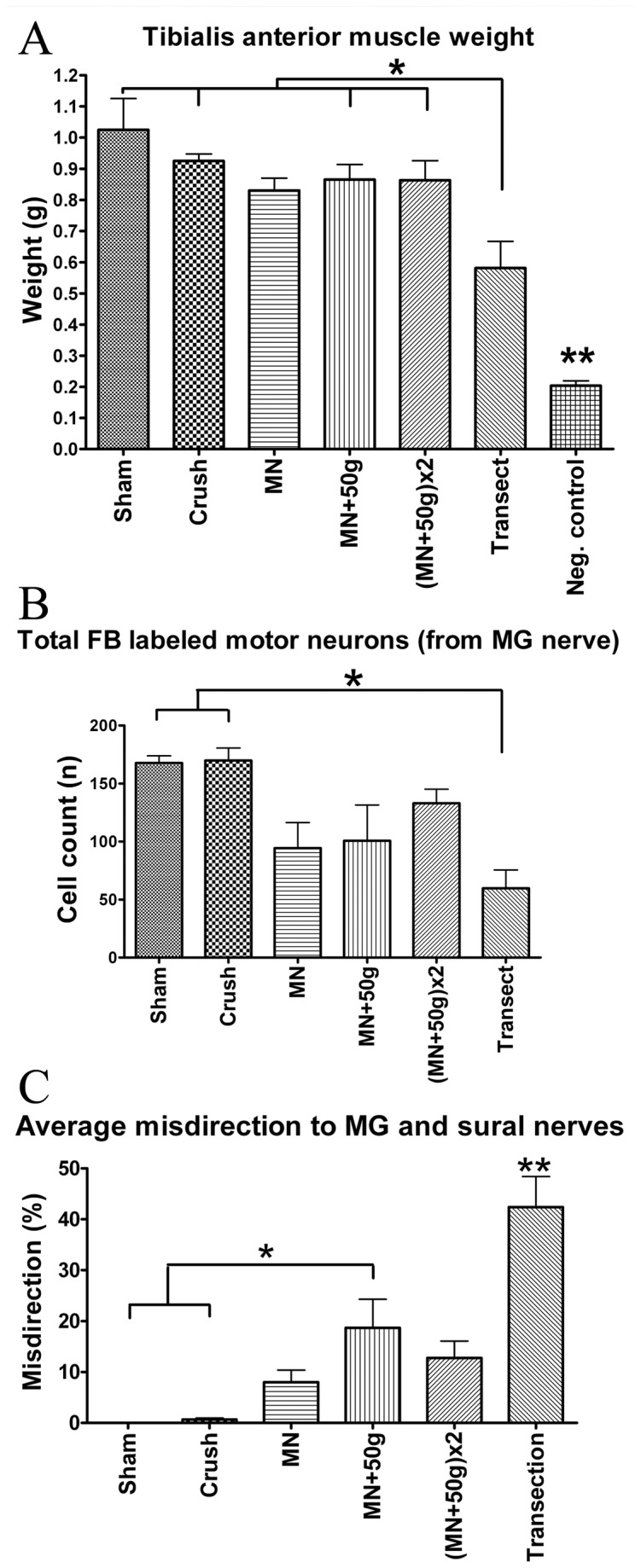
Sciatic experiment muscle weight and motor neuron labeling results. Tibialis anterior muscle weights demonstrated no significant differences between the NIC groups or from *Crush*_***s***_ and *Sham*_***s***_ groups. *Transection*_***s***_ and *Negative* *Control*_***s***_ groups showed statistically significant differences from other groups (A) (n=6 per group). Statistically significant attrition of motor neurons with axons that regenerated into the MG nerve was demonstrated in the *Transection*_***s***_ group, compared to *Sham*_***s***_ and *Crush*_***s***_ groups (B) (n=6 per group). Relative percentage of axonal misdirection to the MG and sural nerves were calculated by dividing the counted cells “out”-side the reference boundaries by the “total” counts (e.g. %misdirection to sural = Di-I-out/Di-I-total x100). *Transection*_***s***_ (n=5) injuries demonstrated the most misdirection and the same trend was found among the NIC injury groups with MG and sural nerve assessments. Average of MG and sural results shown (C) (n=6 per other groups). (*p<0.05,**p<0.001).

In the femoral nerve experiments quadriceps muscles were not weighed because these muscles were denervated by the labeling technique. In the sciatic nerve experiments only selected muscles (plantar and MG) were denervated, which left the tibialis anterior muscle available for assessment. As expected, the tibialis anterior wet muscle weight demonstrated statistically significant differences with heaviest muscles in the ***Sham*_*s*_** (1.03 ± 0.1g), and lowest in the ***NegativeControl*_*s*_** (0.2 ± 0.02g) group (*F*(6, 35)=20.76, *p*<.0001) (Table 2). The ***NegativeControl*_*s*_** group was different from all other groups (*p*<.001) and the ***Transection*_*s*_** group was different from all but the ***MN*_*s*_** group (*p*<.05) on the *post hoc* Tukey’s test (Figure 6A). The mean distance between the two marking sutures (initially 1mm apart) in the ***Transection*_*s*_** group at the time of muscle harvest was 8.89mm (range 6.74 - 10.56mm).

**Table 2:** Sciatic nerve experiment muscle weight, retrograde labeling and final functional results.

**Group (sciatic)**	**Sham_s_**	**Crush_s_**	**MN_s_**	**MN+50g_s_**	**(MN+50g)x2_s_**	**Transection_s_**	**Negative Control_s_**
**Tibialis Anterior (g)**	1.03 ± 0.1	0.93 ± 0.02	0.83 ± 0.04	0.87 ± 0.05	0.86 ± 0.06	0.58 ± 0.09	0.2 ± 0.02
**Fast Blue Total**	167.7 ± 6.16	169.83 ± 10.73	94.33 ± 21.92	100.7 ± 30.88	133 ± 12.24	59.67 ± 15.77	0
**Fast Blue “out”**	0	1.17 ± 0.54	4.17 ± 1.22	9.5 ± 5.03	11.33 ± 4.86	19.6 ± 3.75	N/A
**MG Misdirection (%)**	0	0.73 ± 0.33	6.26 ± 2.03	18.1 ± 8.48	9.98 ± 4.33	30.57 ± 6.5	N/A
**Di-I Total**	90 ± 14.67	82.5 ± 13.96	84.5 ± 14.69	124.8 ± 18.24	104.8 ± 18.04	100.2 ± 35.24	0
**Di-I “out”**	0	0.5 ± 0.34	8.17 ± 4.45	23.67 ± 11.68	19.33 ± 9.75	65.8 ± 21.71	N/A
**Sural Misdirection (%)**	0	0.6 ± 0.48	9.7 ± 4.39	19.21 ± 8.26	15.55 ± 5.13	54.2 ± 7.11	N/A
**Average Misdirection (%)**	0	0.66 ± 0.28	7.98 ± 2.36	18.65 ± 5.64	12.77 ± 3.31	42.39 ± 6.01	N/A
**12-week Slip Ratio (%)**	1.04 ± 1.04	3.89 ± 1.14	27.8 ± 7.03	29.61 ± 13.13	14.17 ± 4	61.07 ± 4.79	56.9 ± 8.06

### Variable attrition and misdirection were found in sciatic NIC groups

All spinal cord specimens of the sciatic nerve experiments were suitable for blinded evaluation of all the serial longitudinal sections. Very few double-labeled cells were detected. One of the ***Transection*_*s*_** group cords had no visible FB or Di-I and misdirection could not be calculated for this animal due to poor regeneration consistent with poor recovery in this animal (54% slip ratio at week 12; muscle weight 0.19g). Di-I labeling was too faint and inadequate for quantification in one animal from the ***Sham*_*s*_** group. 

Both the MG and smaller sural motor neuron pools were well defined in the ***Sham*_*s*_** group with an average number of 167,66 ± 6.16 (FB) and 90 ± 14.67 (Di-I) labeled cells counted, respectively. In the ***Crush*_*s*_** group, total counts were similar and only a few cells were located outside the MG (1.17 of 169.83 ± 10.73) and sural (0.5 of 82.5 ± 13.96) pool boundaries (Table 2). Total FB labeled (MG nerve) motor neuron counts differed significantly between the groups (*F*(5, 30)=5.79, *p*=.0007) (Figure 6B). The three NIC injury groups had variable FB counts, with means between that of the ***Crush*_*s*_** and ***Transection*_*s*_** groups. The ***Transection*_*s*_** group had the lowest number of FB labeled motor neurons (69.66 ± 15.77), different from the ***Sham*_*s*_** and ***Crush*_*s*_** groups (*p*<.01). No statistically significant differences of total motor neurons labeled from the sural nerve (Di-I) were demonstrated between groups (*F*(5, 29)=0.58, *p*=.71) (Table 2).

The average percentage misdirection were also significantly different between groups (*F*(5, 64)=17.48, *p*<.0001) (Figure 6C). Misdirection was variable and intermediate in the NIC groups, but only the ***MN*+50*g*_*s*_** group showed statistically significant differences from the ***Sham*_*s*_** and ***Crush*_*s*_** groups on the *post hoc* Tukey’s test (*p*<.01) (Table 2). ***Transection*_*s*_** group cords demonstrated the greatest degree of disruption of the MG and sural pool organization (Figure 2). The ***Transection*_*s*_** group also had the highest misdirection (30.57 ± 6.5% to MG and 54.2 ± 7.11% to sural) with average misdirection different from all other groups (42.39 ± 6.01%; *p*<.001). As expected, no FB or Di-I was visible in any of the ***NegativeControl*_*s*_** spinal cords.

### NIC injuries had variable recovery, but unrepaired transection injuries did not recover significant function

**Figure 7:**
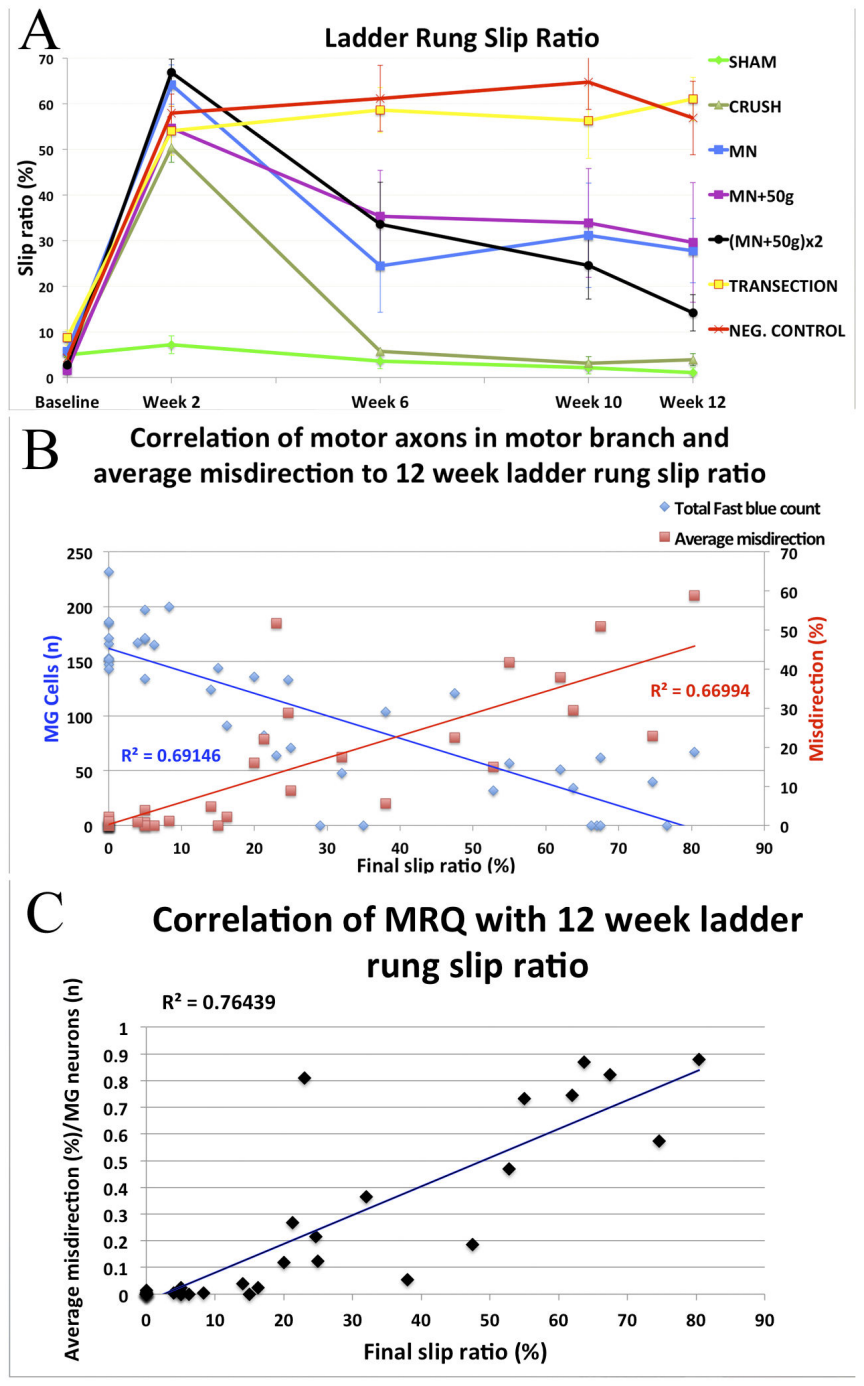
Behavioral results and correlations with axonal misdirection and attrition. In sciatic nerve groups, skilled locomotion was assessed with the ladder-rung task by determining injured limb slip ratios up to 12 weeks. The *Crush*_***s***_ group recovered to baseline, but the *Transection*_***s***_ and *Negative* *Control*_***s***_ groups showed no significant recovery. Despite variable deficits ranging from minimal to extreme in the NIC injury groups, the *MN*_*s*_ and (MN+50g)***x2***_***s***_ groups still demonstrated statistically significant differences from *Crush*_***s***_ at 12 weeks (A) (n=6 per group). More significant than the group results, were the good correlation between individual animal final functional outcomes and the average percentage of misdirection (r^2^= 0.67) and FB labeled motor neuron count (attrition of motor neurons with axons to MG nerve) (r^2^= 0.69) respectively (B). A better correlation was found between the final functional deficit and the misdirection/regeneration quotient (MRQ = Average misdirection (%)/ MG motor neurons (n); r^2^=.76), which combines the relative contribution of both these factors (C).

To assess the integrated motor and sensory behavioral recovery of the injured right hind limbs, ladder rung slip ratios (%) were calculated as the number of right hind limb slips per total number of right hind limb steps at baseline, weeks 2, 6, 10 and 12 (Figure 7A). Consistent with previous reports, the baseline slip ratio was 4.43% with no significant differences between the groups (*F*(6, 35)=1.87, *p*=.50)[15]. At week 2, slip ratios were significantly different (F(6, 35)=25.43, *p*<.0001), as all six groups with nerve injuries made more errors than the ***Sham*_*s*_** group. By week 6, the ***Crush*_*s*_** group slip ratios had returned to pre-injury baseline and ***Sham*_*s*_** group performance. The ***NegativeControl*_*s*_** and ***Transection*_*s*_** groups showed no significant recovery in slip ratio following the injuries (*F*(3, 5)=0.62, *p*=.61 and *F*(3, 5)=1.07, *p*=.39 respectively). Statistically significant differences between groups were present at weeks 6 (*F*(6, 35)=9.82, *p*<.0001), 10 (*F*(6, 35)=9.19, *p*<.0001) and 12 (*F*(6, 35)=12.18, *p*<.0001). The *individual* animal deficits in all three NIC injury groups were highly variable but group performance recovered to slip ratios intermediate between that of ***Crush*_*s*_** and ***Transection*_*s*_** groups. The ***MN*_*s*_** group differed significantly from ***Transection*_*s*_** and ***NegativeControl*_*s*_** groups at week 6 and from ***Transection*_*s*_** at week 12 (*p*<.05). Slip ratios of the ***MN*+50*g*_*s*_** group was significantly different from ***Transection*_*s*_** at week 12 (*p*<.05). The (***MN+50g***)***x2*_*s*_** group differed with statistical significance from ***NegativeControl*_*s*_** at weeks 10 and 12 (*p*<.05; *p*<.01), and from ***Transection*_*s*_** at week 12 (*p*<.001). At the final 12-week assessment, there was still no statistically significant difference between slip ratios of the ***NegativeControl*_*s*_** and ***Transection*_*s*_** groups (Table 2).

### Behavioral outcomes correlated well with axonal regeneration and misdirection

We successfully bridged the gap between minimal (crush) and extreme (transection) injuries with intermediate and variable NIC injuries. This made us more confident to assess the linear correlations between final functional outcome and retrograde labeling. There was a clear linear correlation between the individual animal paired 12-week slip ratios and average misdirection data (r(33)=.82, p <.0001) with r^2^=.67 (Figure 7B). Individual animal 12-week slip ratios also showed a strong negative correlation with the total number of motor neurons projecting into the major motor nerve (MG) sampled (r(39)=-0.83, p<.0001) with r^2^=.69. The combined effect of these two factors (both dependent on injury severity) should yield a better correlation with functional outcome. Indeed, the final functional deficit correlates better with the misdirection/ regeneration quotient (MRQ) (= Average misdirection (%)/ MG motor neurons (n); r^2^=.76), which combines the relative contribution of both these factors (Figure 7C).

## Discussion

### Revisiting nerve injury classification

**Figure 8:**
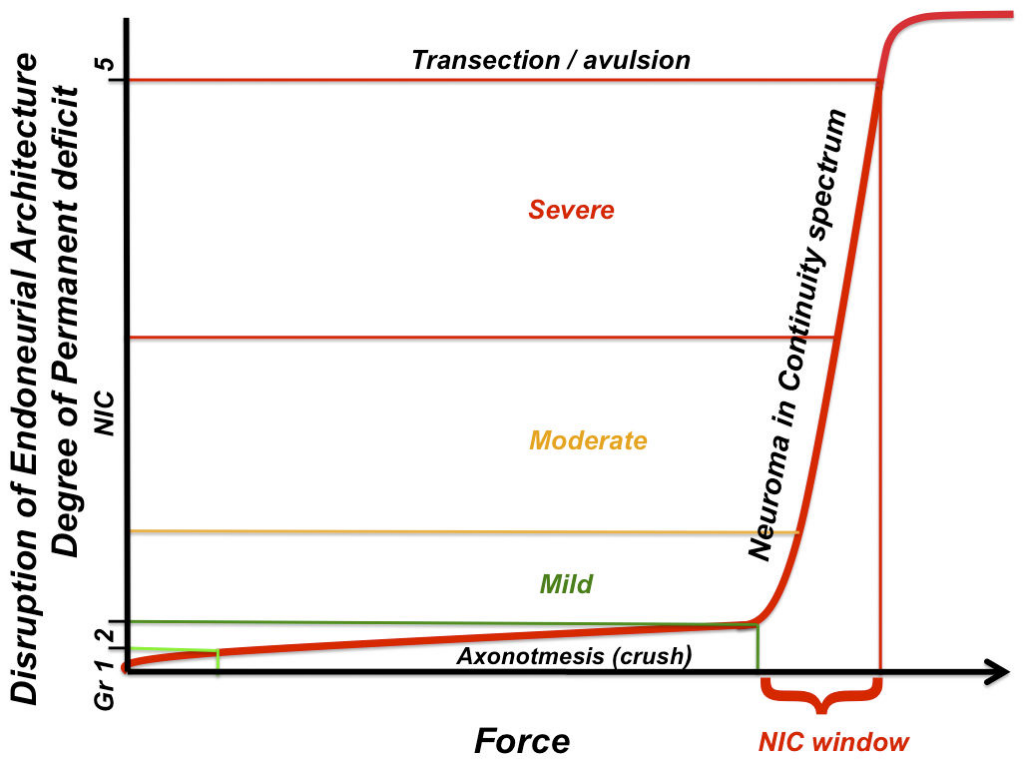
The applied force to severity of nerve injury relationship demonstrating the NIC spectrum and NIC window. Based on the data presented, we propose that the degree of functional deficit that follows traumatic peripheral nerve injuries is dependent on the disruption of the internal nerve architecture of a particular nerve. Sunderland grade 3 and 4 injuries are replaced by the NIC spectrum. Minor variations of force on the hypothetical steep slope within the NIC window may have a dramatic effect on the injury severity and resultant functional recovery.

Based on our findings, we propose that the spectrum of chronic functional deficit between axonotmetic and neurotmetic injuries is strongly related to the degree of disruption of the intrafascicular architecture (Figure 8). This may be focal or cumulate over a distance, depending on the injury mechanism, and lead to proportional axonal attrition and misdirection as major determinants of functional recovery. The original composition of the nerve and associated axotomy and denervation effects related to the location of the injury further compounds to limit functional recovery [20–22]. Although loss of perineurial integrity (according to the Sunderland classification) may be associated with more severe injuries, disruption of this layer *per se* has not been confirmed to be a strong independent determinant of functional recovery following peripheral nerve injuries. For this reason we prefer to group Sunderland Grade 3 and 4 injuries together in the spectrum of NIC injuries. This spectrum will also accommodate the Sunderland mixed and Mackinnon grade 6 injuries in continuity, and given this our experimental model has considerable clinical relevance [6,23]. For an excellent review on the potential mechanisms behind axonal misdirection, we refer the reader to a paper by Allodi et al [29].

### Experimental NIC injuries remain elusive

Despite the employment of three injury paradigms, the recreation of NIC injuries with uniform functional deficits remains challenging. There are apparently additional uncontrolled factors that affect the injury severity, as minor influences may have a dramatic effect at the steep part of the curve (Figure 8). This significantly mimics what is seen clinically, where we are still unable to determine the degree of nerve injury (in-continuity injuries beyond axotomy) without intentional deferral for 3-6 months, to await signs of regeneration. For lack of superior alternatives, this approach is still the standard of practice, despite the recognized detrimental effects of prolonged axotomy and deneravation [20–22]. 

 The addition of traction to the compression force is not essential to recreate experimental NIC injuries. Force settings were selected based on the data available at the time to estimate the “NIC window” between crush and transection with compression force application (Text S1). This “NIC window” was estimated based only on the influence of nerve size on injury thresholds. We have since determined that (not surprisingly) nerve size is not the only determinant of the injury thresholds (Figure S3). With the double injuries (***(MN+50g***)***x2***)**_*f*_**), we have shown that this NIC model is capable of exerting a potential range of injury severity, resulting in attrition and misdirection similar to that of Sunderland Grade 5 injuries in the femoral nerve experiments. We were however unable to replicate the distinction between the outcome of the NIC injury paradigms seen in the femoral nerve experiments, in the sciatic nerves. In fact, the double sciatic injury group (***(MN+50g***)***x2*_*s*_**) appear to have performed better than the other two NIC groups. This supports the notion that there are additional uncontrolled intrinsic differences between these nerves. Nevertheless, the variability of the injuries made it possible to assess correlations with more confidence.

The rats in the ***Transection*_*f*_** group successfully regenerated transected femoral nerve fibers across an average distance of 3.45mm without any form of repair and performed similar to the ***Transection+Repair*_*f*_** group in most aspects. This emphasizes the resilience of these animals, compared to humans, in which little if any spontaneous distal reinnervation is expected without some form of repair [6,7]. However, only a relatively small gap needed to be bridged. Intersestingly, the only advantage that the ***Transection+Repair*_*f*_** group showed above ***Transection*_*f*_** in this study was a significant decrease in axonal misdirection, which correlated strongly with functional recovery in the second part of this study. We therefore speculate that additional functional outcome measures for the femoral nerve experiments would likely have demonstrated some additional benefit in the ***Transection+Repair*_*f*_** over the ***Transection*_*f*_** group.

### Assessment of axonal misdirection can be challenging

Our neuron counts (not corrected for double counting) compare well to other published reports, although they are mainly intended for inter-group comparison, not to reflect the absolute numbers [24,25]. For both femoral and sciatic experiments, the labeling results are presented as the relative proportions of motor neurons with incorrectly directed axons. Some motor division projections may still be along sensory (e.g. proprioceptive) pathways and technically also incorrect. Therefore, in the femoral nerve experiments, all the FB-labeled motor neurons cannot be assumed to have correctly directed axons, but the Di-I labeled neurons certainly have misdirected projections. Similarly, many of the “in” counts of the sciatic experiments may represent motor neurons of overlapping (foreign) pools.

Misdirection onto the sural nerve was more apparent than in the MG sample. This may be because the sural nerve motor neuron pool rostral boundary is more caudal in the sciatic pool, which would increase the likelihood of detecting motor neurons with axons misdirected into this nerve with the methods used. The sural nerve is also primarily a sensory nerve with smaller proportion of motor axons compared to the MG nerve [26]. If initial regeneration is random, a comparatively greater proportion of motor axons may be directed into this nerve, with less apparent attrition [27]. As we used the sural and MG nerves as convenient samples to assess relative misdirection, and because the results of the two showed the same trend, we used the average results of the two samples for further correlations. Similar to the femoral nerve experiments, we regarded the attrition of motor neurons with projections into the major motor (MG) nerve assessed, to give the best representation of the fate of efferent axons in the sciatic experiments.

### Functional deficit and correlations

A recovery period of 4 weeks has been shown to be sufficient to assess misdirection in the femoral nerve model [25]. A period of 12 weeks was allowed in the sciatic nerve experiments to ensure that functional recovery has plateaued [14,28]. We only used one functional outcome measure, as the ladder rung task is a sensitive test that provides reliable results in our hands. Others have confirmed that it is one of several skilled locomotion tasks that provide excellent resolution of integrated motor and sensory recovery of hind limb function following sciatic nerve injuries [18]. However, confirmation of our results with additional outcome measures is needed to support our findings. Interestingly, the ***Transection*_*s*_** group showed no functional recovery with the measure we used, concordant with the Sunderland classification, despite significant recovery of muscle mass after spontaneous bridging of an average 8.8mm gap in five of six animals. This emphasizes the detrimental effect of axonal misdirection on functional recovery in mixed motor nerve injuries, despite successful muscle reinnervation.

Brushart comprehensively reviewed the literature for experimental outcome measures that correlate with measures of functional recovery and found the evidence for direct correlation lacking [4]. Wikholm et al. provided data to suggest a good correlation between the degree of axonal misdirection and functional recovery (Sciatic Functional Index) following repair of experimental sciatic nerve injuries, although such a correlation was not presented in their paper [28]. After common peroneal nerve transection and repair, Wasserschaff demonstrated good correlation between disturbance of the tibialis anterior pool topography, cell count and incoordination between tibialis anterior and MG EMG activity in walking mice [30]. Direct functional analysis was not performed in that study. Valero-Cabré and Navarro have demonstrated similar correlations between surrogate misdirection estimations (by CMAP amplitude) with walking track outcomes by using sciatic nerve transection injuries with different repair strategies [8]. The results used in these correlations for the most part, do not bridge the gap between minimal and maximal deficits [4]. The choice of functional outcome measures may also be critical, as complex compensatory behavioral changes may mask the effect of axonal misdirection [31,32]. 

To our knowledge, this is the first time more direct motor axon misdirection and attrition estimations were correlated to functional outcome after clinically relevant experimental peripheral nerve injuries that includes the neuroma-in-continuity injury spectrum. This work emphasizes the need to continue to focus on strategies that promote both robust and accurate nerve regeneration to optimize functional recovery and we anticipate that this NIC model will prove to be of value to more investigators striving to remedy peripheral nerve injuries.

## Supporting Information

Figure S1 **Instrument modification and calibration.** Modified malleus nipper (MN): An adjustable stop was added to the handles of the MN for force adjustment and tip serrations were filed down for uniform force distribution (A). The MN was calibrated with a load cell to estimate the force for each instrument setting (B). The uneven and incomplete cut (arrows) of an unmodified MN on parafilm (C). The clean parafilm cut of the modified MN (D).
(TIF)

Figure S2 **Early signs of NIC in femoral nerve.** A longitudinal section of a femoral nerve injury zone 5 days after ***MN*+50*g*** injury with NF 200 in green to demonstrate the axonal profiles and Rhodamine Phalloidin that highlights the f-actin in yellow to help define the perineurium (white arrows) and small bloodvessels (bold white arrows) (A). Magnification of the box in A for more detail of the neurofilament stained axons (B). Regenerating axons in the interfascicular and extrafascicular compartments are pointed out with red arrows in corresponding areas in A, B, C and D (C and D at higher magnification from boxes in B). 250µm scale bar.
(TIF)

Figure S3 **Transection thresholds and predicted NIC windows.** Average transection threshold pressure varies significantly depending on the target nerve (A). The predicted NIC windows between the upper crush and lower transection thresholds (shaded green) for the femoral (B) and sciatic (C) nerves were estimated with the available data. A single force setting was selected for the MN to cover the size range of the femoral or sciatic nerves (fem: femoral; CP: common peroneal).
(TIF)

Text S1 **Additional findings are reported here to support the methods employed and aid other investigators who may want to employ this model.**
(DOC)
