# Supplementary figures and images for: The Impact of Motor Axon Misdirection and Attrition on Behavioral Deficit Following Experimental Nerve Injuries
Source: PLoS One. 2013 Nov 25;8(11):e82546. doi: 10.1371/journal.pone.0082546 (PMC3839879; doi:10.1371/journal.pone.0082546)

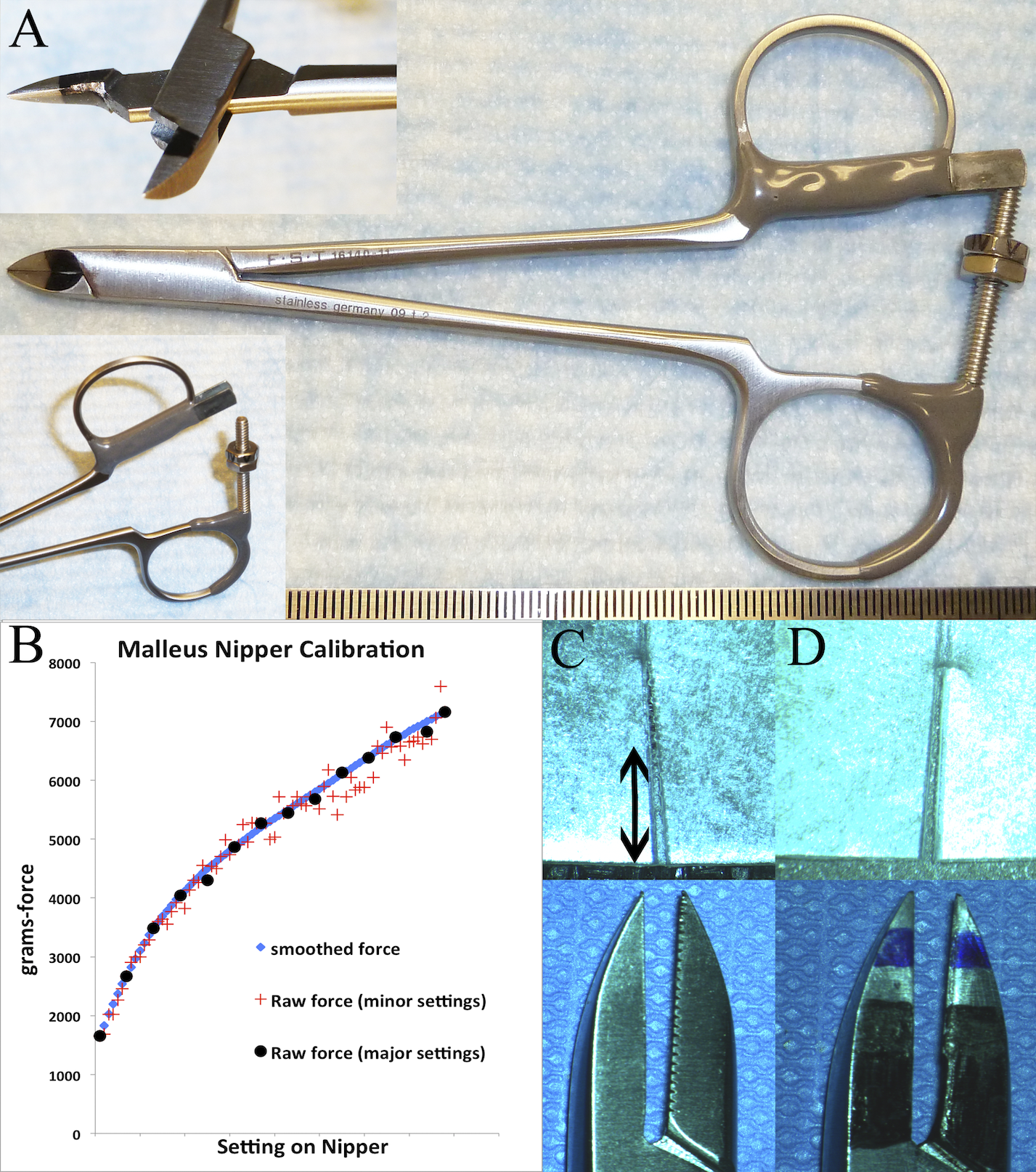

Supplement: Figure S1 — Instrument modification and calibration. Modified malleus nipper (MN): An adjustable stop was added to the handles of the MN for force adjustment and tip serrations were filed down for uniform force distribution (A). The MN was calibrated with a load cell to estimate the force for each instrument setting (B). The uneven and incomplete cut (arrows) of an unmodified MN on parafilm (C). The clean parafilm cut of the modified MN (D). (TIF) [file pone.0082546.s001.tif]

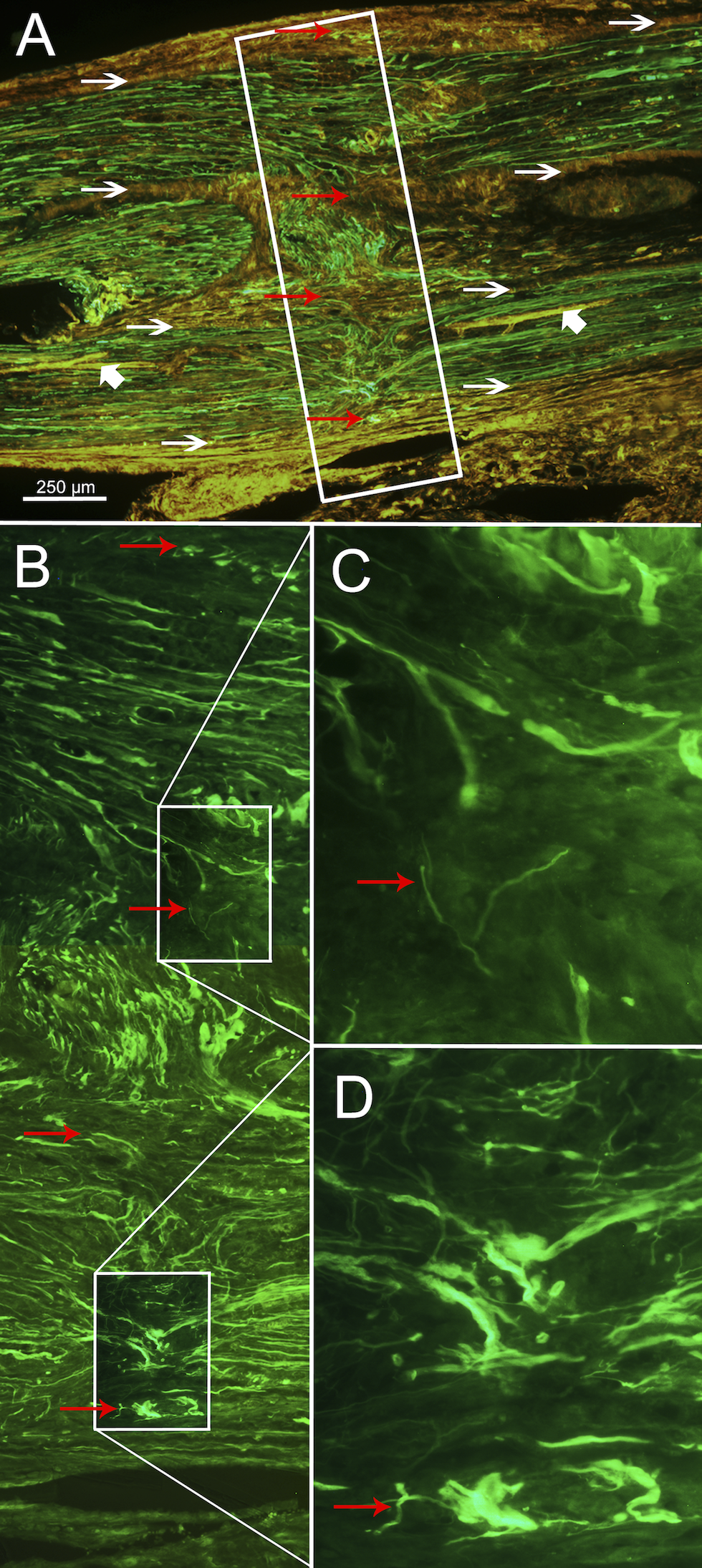

Supplement: Figure S2 — Early signs of NIC in femoral nerve. A longitudinal section of a femoral nerve injury zone 5 days after MN+50g injury with NF 200 in green to demonstrate the axonal profiles and Rhodamine Phalloidin that highlights the f-actin in yellow to help define the perineurium (white arrows) and small bloodvessels (bold white arrows) (A). Magnification of the box in A for more detail of the neurofilament stained axons (B). Regenerating axons in the interfascicular and extrafascicular compartments are pointed out with red arrows in corresponding areas in A, B, C and D (C and D at higher magnification from boxes in B). 250µm scale bar. (TIF) [file pone.0082546.s002.tif]

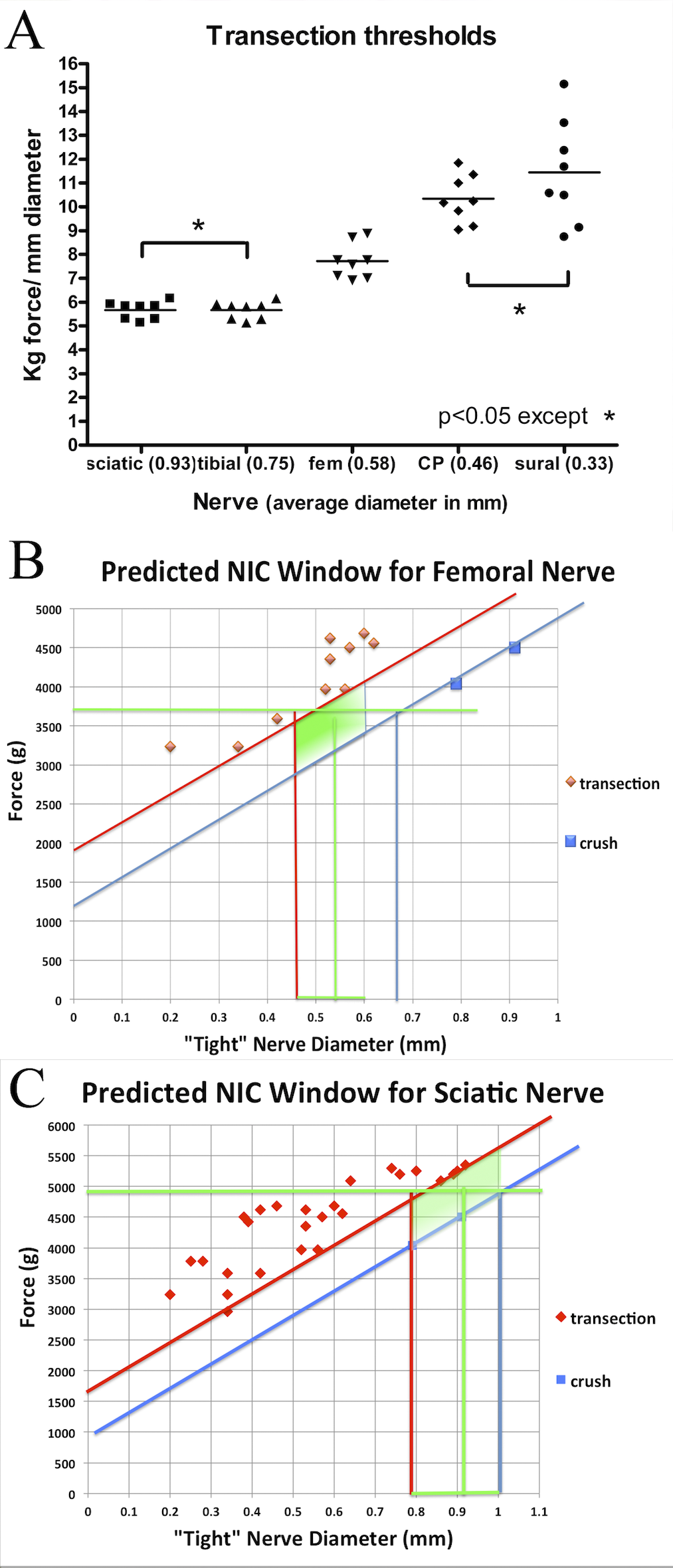

Supplement: Figure S3 — Transection thresholds and predicted NIC windows. Average transection threshold pressure varies significantly depending on the target nerve (A). The predicted NIC windows between the upper crush and lower transection thresholds (shaded green) for the femoral (B) and sciatic (C) nerves were estimated with the available data. A single force setting was selected for the MN to cover the size range of the femoral or sciatic nerves (fem: femoral; CP: common peroneal). (TIF) [file pone.0082546.s003.tif]
