# Supplementary material for: The Impact of Motor Axon Misdirection and Attrition on Behavioral Deficit Following Experimental Nerve Injuries
Source: PLoS One. 2013 Nov 25;8(11):e82546. doi: 10.1371/journal.pone.0082546 (PMC3839879; doi:10.1371/journal.pone.0082546)
Supplement: Text S1 — Additional findings are reported here to support the methods employed and aid other investigators who may want to employ this model. (DOC) [file pone.0082546.s004.doc]

**Supporting Text S1: Additional findings are reported here to support the methods employed and aid other investigators who may want to employ this model.**

***Instrument refinement and “Parafilm test”:***

The MN that was fitted with an adjustable stop enabled calibration with a thin load cell (Flexiforce, medium B201 with ELF™, Tekscan Inc., South Boston, MA, USA) (Figure S1). Even distribution of pressure by the MN onto the nerve was found to be important. Without this, part of the nerve may be transected while another only crushed, confounding the objective of the NIC model to selectively disrupt the internal nerve architecture without disrupting gross continuity of the epineurium. For this reason serrations on the MN were filed down. Measuring the true pressure proved difficult, as pressure is a function of force over surface area. Due to the lever arm effect, the force exerted by the most distal blade tips of the instrument, will be lower than at a more proximal location in the blades. This can be overcome by slightly reducing the contact surface distally, increasing the effective local pressure.

Rather than measuring the precise pressure, the effect of the applied force was easier to evaluate. We found that simple laboratory film (Parafilm “M”, Bemis Flexible Packaging, Oshkosh, WI, USA) was useful for this. At any particular force (instrument) setting, the uniformity of the parafilm impression made by the part of the blade where the nerve is to be positioned can be assessed under an operating microscope. This is best demonstrated when the force is increased to where it cuts through the parafilm (Figure S1). The pressure required to cut the parafilm is close to the minimum pressure needed to inflict NIC injury in average rat sciatic nerves. If the instrument is not capable of cutting through parafilm (without lateral shear), it is unlikely that the desired injury will be inflicted, especially in larger nerves. If this is the case, the blade edges may be further sharpened to increase the effective local pressure. Lateral play in the instrument should be corrected to avoid unpredictable shear injury that may unintentionally transect the target nerve.

***Nerve characteristics and “tight” nerve diameter:***

We found that the nerve caliber is important in selecting an appropriate instrument setting, with a direct relationship to the compression-transection force (Figure S3). Nerve sizes vary between different nerves, along the course of a particular nerve and with the growth of the animal. We were able to demonstrate significant differences in the susceptibility of different nerves to a compression force, after compensation for nerve caliber (Figure S3). Variations in nerve collagen content and fascicular structure may account for this [33]. For experimental purposes we found that it is important to reduce these variables by selecting a precise injury zone, away from branching points.

The caliber of small and pliable nerves *in vivo* is difficult to measure objectively. To improve consistency in measurements, the “tight” nerve diameter was used, as measured with an electronic micro-caliper. Gentle traction is applied to the nerve to be measured by gently hooking it with an instrument. This reduces the nerve diameter slightly, but makes the nerve surface less pliable for easier measurement. The nerve transection thresholds (instrument setting) for particular nerves at specified locations were plotted against nerve size in pilot experiments (Figure S3). With this information and the “tight” nerve diameter range of the experimental groups, sub-transection settings were selected aiming to reproduce NIC injuries in the groups of animals.

***NIC window:***

The selection of the maximal sub-transection compression force may be critical since the “NIC window” between simple crush and transection is quite small, as the transition from crush to NIC occurs close to the transection threshold (Figure S3). Histological features of crush injury can however be reproduced using a wide range of forces [34,35]. Because of inter-animal variation, a high force needs to be selected to reduce contamination of the NIC group with effective crush injuries. To distinguish between crush and NIC using histological features is difficult before 5 days post injury. This short delay allows axons to regenerate across the injury zone to reveal aberrant regeneration patterns that support distortion of the endoneurial and perineurial architecture (Figure S2).

***Motor neuron labeling****:*

We elected to use the simultaneous retrograde labeling technique in all the experiments. Di-I and FB are reliable dyes that yield consistent results and emit distinct and easily distinguishable signals (yellow and blue respectively) [36]. Di-I takes longer than FB to be retrogradely transported [37]. In pilot experiments we found brighter Di-I labeling with more time (13 days) allowed after application. The crystal-in-cap method logistically simplifies dye application. Caps are prefilled with dye crystals so that nerves can be transected, capped and wounds closed much quicker compared to “well” application where nerve endings are typically exposed for as long as an hour [38]. This reduces surgical time and anesthetic exposure to the animals significantly. We found the well and injection techniques also technically difficult to use on small (and short) caliber nerves in confined surgical exposures without risking dye contamination to the field. The crystal-in-cap method however does risk dye leakage (leading to the exclusion of one animal from analysis in this study), especially if the silicone caps are too big (internal diameter). Alternative sequential labeling techniques introduce additional injury and dye spread after injection may be variable. For sequential labeling, dye toxicity, fading and persistence also need to be considered for a 12-week study [39,40].

***Sciatic motor neuron misdirection:***

The sciatic nerve contains a mixture of afferent and efferent axons innervating functionally antagonistic muscles. Normally, the motor neurons of individual muscles are very elegantly grouped within the ventral horn grey matter [24]. The major peroneal (fibular) and tibial motor neuron pools overlap significantly as each span up to 2/3 of the length of the sciatic pool [24]. Because the somatotopical organization of motor neurons labeled from individual distal nerve branches would be disturbed as a result of axonal misdirection, accurate discrimination between smaller motor neuron pools becomes difficult, as organizational boundaries between adjacent and overlapping pools are lost. Despite these difficulties, previous investigators have successfully used techniques based on the motor neuron pool topography and relative compound muscle action potential contributions to assess axonal misdirection after experimental sciatic nerve injuries [1,8,41,42].

The sampling technique we used was based on the topography of smaller motor neuron pools, organized most caudally within the greater sciatic nerve pool. The MG nerve (branch of the sciatic) contains projections of only approximately 145 of the ±2000 sciatic motor neurons [24,43]. Although the sural nerve is primarily a sensory nerve, in rats it also contains axons from approximately 71±22 motor neurons, which innervate plantar muscles [24,44]. These smaller MG and sural nerve pools span the most caudal 50% or less of the total sciatic pool [45]. We used the most caudal extent of the sciatic pool as a fixed reference point to which the longitudinal distribution (rostro-caudal span) of labeled neurons could be referenced. Quantification of the motor neurons labeled from these nerves that lie outside (rostral to the rostral reference) of their normal longitudinal boundaries, provide a conservative but reliable way to compare the relative misdirection between groups and individual injuries. Lateral overlap with other pools was disregarded.

**References:**

33. Sunderland S (1978) Nerves and Nerve injuries*.* 2^nd^ ed. Edinburgh : Churchill Livingstone. pp. 38-45.

34. Beer GM, Steurer J, Meyer VE (2001) Standardizing nerve crushes with a non-serrated clamp. J Reconstr Microsurg 17: 531-534.

35. Ronchi G, Raimondo S, Varejão AS, Tos P, Perroteau I, *et al*. (2010) Standardized crush injury of the mouse median nerve. J Neurosci Methods 188: 71-75.

36. Kobbert C, Apps R, Bechmann I, Lanciego JL, Mey J, *et al*. (2000) Current concepts in neuroanatomical tracing. Prog Neurobiol 62: 327-351.

37. Choi D, Li D, Raisman G (2002) Fluorescent retrograde neuronal tracers that label the rat facial nucleus: a comparison of Fast Blue, Fluoro-ruby, Fluoro-emerald, Fluoro-Gold and DiI. J Neurosci Methods 117: 167**-**172.

38. Al-Majed A, Neumann CM, Brushart TM, Gordon T (2000) Brief electrical stimulation promotes the speed and accuracy of motor axonal regeneration. J Neurosci 20: 2602-2608.

39. Novikova L, Novikov L, Kellerth JO (1997) Persistent neuronal labeling by retrograde fluorescent tracers: a comparison between Fast Blue, Fluoro-Gold and various dextran conjugates. J Neurosci Methods 74: 9-15.

40. Puigdellivol-Sanchez A, Prats-Galino A, Ruano-Gil D, Molander C (2003) Persistence of tracer in the application site - a potential confounding factor in nerve regeneration studies. J Neurosci Methods 127: 105-110.

41. Brushart TM, Mesulam MM (1980) Alteration in connections between muscle and anterior horn motoneurons after peripheral nerve repair. Science 208: 603-605.

42. English AW (2005) Enhancing axon regeneration in peripheral nerves also increases functionally inappropriate reinnervation of targets. J Comp Neurol 490: 427-441.

43. Peyronnard JM, Charron LF, Lavoie J, Messier JP (1986) Motor, Sympathetic and Sensory Innervation of Rat Skeletal Muscles. Brain Res 373: 288-302.

44. Peyronnard JM, Charron LF, Lavoie J, Messier JP (1986) Differences in horseradish peroxidase labeling of sensory, motor and sympathetic neurons following chronic axotomy of the rat sural nerve. Brain Res 364: 137-150.

45. Nicolopoulos-Stournaras S, Iles JF (1983) Motor Neuron Columns in the Lumbar Spinal Cord of the Rat. J Comp Neurol 217: 75-85.
